# Mapping of 30-meter resolution tile-drained croplands using a geospatial modeling approach

**DOI:** 10.1038/s41597-020-00596-x

**Published:** 2020-08-05

**Authors:** Prasanth Valayamkunnath, Michael Barlage, Fei Chen, David J. Gochis, Kristie J. Franz

**Affiliations:** 1 National Center for Atmospheric Research (NCAR), Boulder, Colorado 80301 USA; 2 Geological and Atmospheric Sciences, Iowa State University, Ames, Iowa 50011 USA

**Keywords:** Environmental impact, Hydrology

## Abstract

Tile drainage is one of the dominant agricultural management practices in the United States and has greatly expanded since the late 1990s. It has proven effects on land surface water balance and quantity and quality of streamflow at the local scale. The effect of tile drainage on crop production, hydrology, and the environment on a regional scale is elusive due to lack of high-resolution, spatially-explicit tile drainage area information for the Contiguous United States (CONUS). We developed a 30-m resolution tile drainage map of the most-likely tile-drained area of the CONUS (AgTile-US) from county-level tile drainage census using a geospatial model that uses soil drainage information and topographic slope as inputs. Validation of AgTile-US with 16000 ground truth points indicated 86.03% accuracy at the CONUS-scale. Over the heavily tile-drained midwestern regions of the U.S., the accuracy ranges from 82.7% to 93.6%. These data can be used to study and model the hydrologic and water quality responses of tile drainage and to enhance streamflow forecasting in tile drainage dominant regions.

## Abstract (summary)

**Table:** 

Measurement(s)	30-meter resolution tile drainage map • spatial pattern
Technology Type(s)	digital curation • computational modeling technique
Sample Characteristic - Environment	area of cropland
Sample Characteristic - Location	contiguous United States of America

Machine-accessible metadata file describing the reported data: 10.6084/m9.figshare.12668234

## Background & Summary

Subsurface tile drainage is one of the most widely-used agriculture management practices to enhance crop yield in regions with high water tables or poorly drained soils. Based on the U.S Department of Agriculture (USDA) National Agriculture Statistics Service (NASS) 2017 Census of Agriculture^1^, the spatial extent of tile-drained croplands in the United States is around 22.48 million ha, out of which 18.79 million ha (83.8%) are mainly in six Midwestern states. Tile drains, generally installed below the crop rootzone to drain excess water from the soil, improve soil aeration and soil quality in the rootzone^2^, and enhance crop yield^2,3^. Compared to undrained cropland, tile drainage also causes significant changes in watershed hydrology^4,5^. Studies have shown subsurface drainage removes excess water from the rootzone^6^, results in higher infiltration and lower surface runoff^7^, peak flows^8^ and flooding^9^. Tile drainage tends to increase the watershed baseflow^10,11^, decrease groundwater travel times^10,12^, increase annual runoff volume^4,13–16^, and increase instream nitrate concentrations^4,8,17,18^. Subsurface tile drainage can influence the runoff volume, the timing and shape of the hydrograph^4,5^, and the local and regional climate by reducing ET from croplands^19,20^.

Correctly modeling the tile-drainage impacts on the hydrologic cycle is a daunting challenge due to the lack of continental-scale high-resolution tile-drainage data^21^, because farm-, field-, and subcounty-scale information on tile drainage are not available. USDA-NASS has well documented the county-level tile-drained area for the U.S., however, the geographical information on the spatial distribution of tile-drained croplands within each county are not provided. As subsurface tile drainage has significant effects on land surface hydrology^4,5^, water quality^22^, local and regional climate^19,20^, accurate, detailed geographical information on the sub-county spatial distribution of tile drained croplands is indispensable to answer a wide variety of questions related to hydrology, climate, and earth system science^21^.

During the past two decades, several spatial and gridded data of tile drainage have been developed for the U.S. For instance, Jaynes and James^23^ developed gridded (30-m resolution) tile drainage fraction data for the 23 most intensively drained states in the U.S. with a Geographic Information System (GIS) approach using the 1992 National Land Cover Database (NLCD) croplands and Natural Resources Conservation Service (NRCS) State Soil Geographic (STATSGO) poorly drained soil dataset. By applying a similar approach, Sugg^21^ developed tile-drainage data for the Midwestern corn belt of the U.S. using 1992 NLCD row crops information and STATSGO drainage class and soil hydrologic soil groups; however, the result is likely to be an overestimate compared to census data because their method classified all the row crops with poorly drained soils as tile trained. Using a GIS-based analysis, Sui^24^ generated a tile drainage map for Indiana considering land cover, soil, and slope data where the slope is less than 2%, and soils are poorly drained. By employing aerial imagery, Naz *et al*.^25^ identified tile drainage locations in Hoagland watershed of Indiana. Moreover, Brown^26^ mapped tile drainage locations in three counties of Minnesota with GIS by using croplands from NLCD - 2001 and NASS - 2008, soil drainage class, hydrologic group, and land capability class from SSURGO data, and topographic slope (less than 2%) from National Elevation Data (NED). Nakagaki *et al*.^27^ developed 30-m gridded tile-drainage fraction data for the conterminous U.S. for the year 1992 using NLCD 2011 and STATSGO version 2.0 data using the Sugg^21^ methodology. These data are updated using the 2012 USDA-NASS census but only for 12 Midwestern states^28^. Recently, Cho *et al*.^29^, identified tile-drained croplands of the Red River basin covering portions of North Dakota, South Dakota, and Minnesota using satellite big-data and random forest machine learning approaches. Their data‐intensive machine learning approach indicated that soil properties and land surface temperatures are the strongest predictors of tile drainage. However, this approach is data-, time-, and computationally-intensive, and constrained to the quantity, quality, and resolution of satellite data or predictors. Also, the quality of the tile drainage map generated using this approach relies on the training samples or the quantity and quality of field-level ground-truth information (or tile drainage permit records). Thus, extending this tile drainage mapping approach to other regions or the CONUS scale is a cumbersome task.

Remote sensing-based (e.g. microwave brightness signatures) tile-drainage mapping approaches primarily depend upon the soil reflectance. Soil above the tile drainage tends to dry faster (higher reflectance) compared to the soil midway (low reflectance) between two tile drainage lines. Previous studies have indicated that the ideal time to acquire aerial imagery for tile drainage mapping is within three days after a 25 mm or greater rainfall event^30,31^. That is, the degree of accuracy of a remote sensing approach to identify tile drainage over a large area mainly depends upon the time of acquiring aerial imagery after a rainfall event and presence of vegetation cover^32^. Despite the high performance of remote sensing approaches to delineate tile drainage over a field or watershed scale, mapping tile drainage for regional or national level is expensive and constrained by weather and availability of resources.

To date, comprehensive well-validated, gridded, high-resolution tile drainage data are unavailable for the U.S. Here, we develop a present-day, 30-m resolution tile drainage map for the CONUS using USDA Census of Agriculture 2017 county-level tile drainage information, NLCD-2016 cropland, SRTM-DEM derived slope, SSURGO version 2.0 soil drainage information and by employing a GIS model. We present verification of the resulting tile drainage map with 16000 field-level ground-truth points.

## Methods

This section first discusses the list and descriptions of data required for mapping tile drainage croplands of the U.S. Next, it describes details of a strong yet simple and easy to implement GIS decision tree model to delineate tile drainage areas, hereafter referred as SSURGO SRTM-slope defined Agriculture Tile Drainage Dataset for the U.S. (AgTile-US).

### County tile drainage statistics

**Fig. 1:**
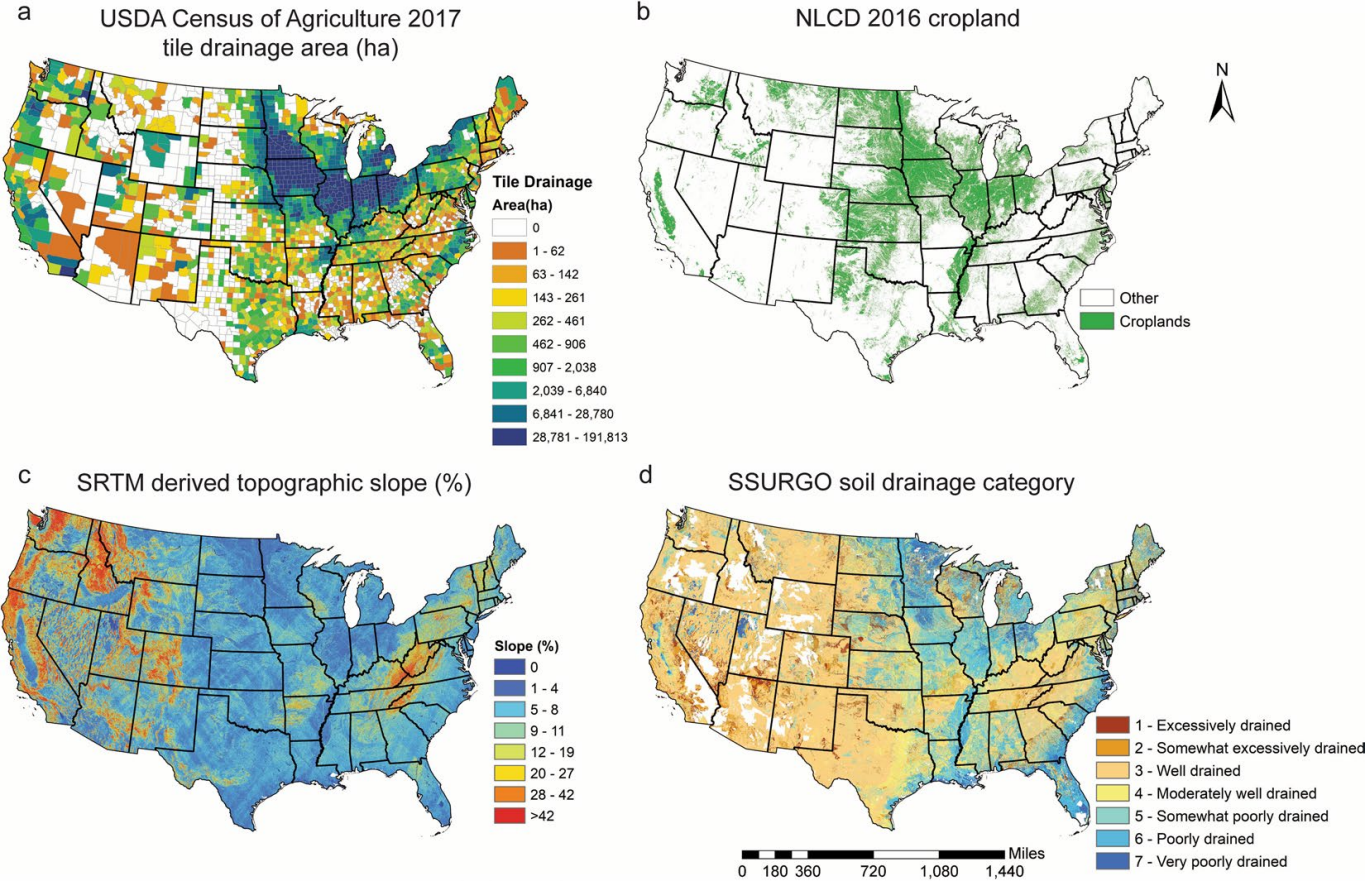
Input data to AgTile-US GIS model for generating gridded tile drainage data for the U.S. (**a**) County-level tile drainage area (ha) from USDA Census of Agriculture 2017, (**b**) National Land Cover Database (NLCD) 2016 cropland mask at 30 m resolution, (**c**) Shuttle Radar Topography Mission Digital Elevation Model derived slope (%) at 30 m, and (**d**) Spatial pattern of soil drainage characteristics based on the SSURGO database at 30 m.

The most critical input into the AgTile-US geospatial model is the county-level tile-drainage census estimates from USDA, which is conducted once every five years. Here, we use the most recent census data (Census of Agriculture, 2017)^1^. It is the best tile drainage area estimate available for the entire U.S. Using the USDA-NASS Quick Stats tool^33^, the tile drainage area information and error estimate were downloaded in spreadsheet format. It was then joined to the U.S. county shapefile in ESRI ArcMap 10.7^34^ before feeding into the AgTile-US geospatial model. The spatial distribution of county-level tile drainage is presented in Fig. 1a. The north-central U.S. shows the greatest areal extent of tile drainage, especially in Iowa (5.7 Mha), Illinois (3.83 Mha), Minnesota (3.27 Mha), Indiana (2.57 Mha), Ohio (2.18 Mha), and Michigan (1.22 Mha). These states are in the headwater regions of the Mississippi River and contribute a significant amount of runoff, sediment, and nutrients to the Mississippi River. Parts of the north-eastern U.S, coastal areas of south-eastern U.S., and the south-central U.S. also show a localized high-intensity tile drainage.

### National land cover database (NLCD) 2016

To constrain the mapping of tile drained areas to cropland, we ingested a cropland mask (Fig. 1b) derived from NLCD 2016^35^ into the geospatial model. Since tile drainage mapping by the geospatial model is mainly based on the county-level census estimate, soil drainage categories, and topographic slope, a cropland mask is necessary to mask out non-agricultural lands. The “cultivated cropland” category from NLCD 2016 was used to define the cropland mask, and cells or grids that were not cropland were masked out of the drainage category raster and slope raster by the geospatial model before mapping the tile drainage areas.

### Slope generated from Shuttle Radar Topography Mission (SRTM) derived DEM

The SRTM^36^ is an international research effort intended to generate a high-resolution (30-m to 90-m) digital topographic database on the near-global scale (56°S to 60°N). This project was a combined effort of the National Aeronautics and Space Administration (NASA) and the National Geospatial-Intelligence Agency (NGA). The data were collected by interferometric radar, which compares two radar signals captured at slightly different angles by two different antennas. Surface elevation was estimated using the differences between the two radar signals. In this study, we used 30-m resolution, void-filled DEM data from SRTM to estimate topographic slope for the CONUS. The data were downloaded as GeoTIFF format from the Opentopography website (https://opentopography.org/; accessed in October 2019). The topographic slope (Fig. 1c) was estimated using ESRI ArcMap10.7 at 30-m resolution.

### Soil Survey Geographic (SSURGO) data from USDA NRCS

The SSURGO database (https://websoilsurvey.nrcs.usda.gov/; accessed on 15 October 2019) contains soil information collected by the National Cooperative Soil Survey of the U.S. The soil information was obtained by field visits and laboratory analysis. The collected soil information is available at scales ranging from 1:12,000 to 1:63,360 in database tables and maps. The SSURGO database contains various information including available water capacity, soil reaction, electrical conductivity, drainage characteristics, soil texture, and frequency of flooding; yields for cropland, woodland, rangeland, and pastureland; and limitations affecting recreational development, building site development, and other engineering uses. Map units are the map outline areas, and within a map unit, soil and other components have unique properties, interpretations, and productivity. Maps can be linked to database tables using the unique map unit number to extract information about component soils and their properties. Information in SSURGO can be viewed in the Web Soil Survey (WSS) or obtained in ESRI shapefile format for the study area.

In this study, we used 10-m resolution, gridded SSURGO (gSSURGO) data obtained for the CONUS from the USDA Geospatial Data Gateway (https://datagateway.nrcs.usda.gov/GDGHome_DirectDownLoad.aspx; accessed on 15 October 2019). The gSSURGO database consists of a 10-m resolution map unit raster in GeoTIFF format (MapunitRaster_10m.tif) along with a component database table. Soil drainage classes are prepared by joining the map unit raster to its component database table in ESRI ArcMap using the map unit key (MUKEY) and the resulting raster consisting of seven drainage classes was then resampled to a 30-m resolution raster. This raster helped the AgTile-US GIS model to identify tile drainage by distinguishing soil with different drainage categories. The spatial pattern of soil drainage information is depicted in Fig. 1d. Most of the poorly drained soil are found in the north-central U.S., parts of the south-central U.S., especially surrounding the Mississippi River, and coastal states of the south-eastern U.S.

### The AgTile-US geospatial tile drainage model

**Fig. 2:**
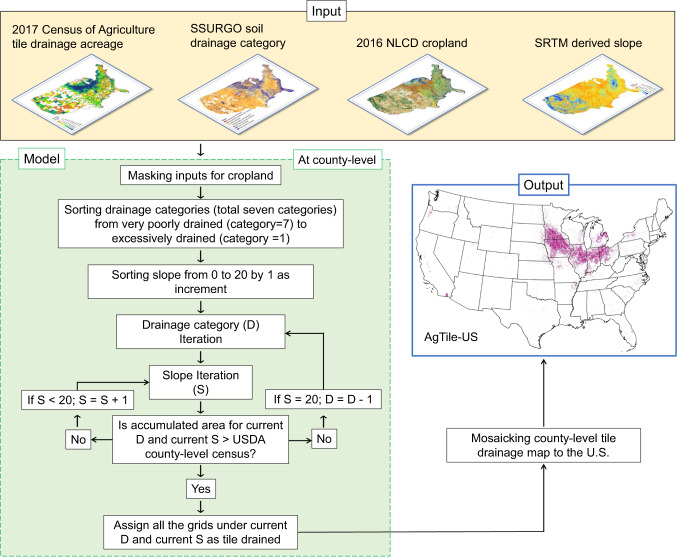
GIS Workflow diagram of the SSURGO SRTM-slope defined Agriculture Tile Drainage Dataset (AgTile-US) methodology. The output map has a spatial resolution of 30 m.

A geospatial model was developed to map tile drainage areas based on soil drainage information and a topographic slope threshold within agricultural land cover that constitute an equivalent tile drainage area by county estimated by the Census of Agriculture 2017 (Fig. 2). The geospatial framework was based on the idea that subsurface tile-drainage systems are needed for flat (or low slope) croplands with poorly drained soil. The model was implemented at county-level and initiated by generating an ordered list of drainage category (D) (see legends of Fig. 1d for seven drainage categories and corresponding numbers) and slope (S). To validate the AgTile-US and to set the upper limit of slope in the geospatial model, we have manually identified 16000 ground-truth points of tile drainage across the U.S. using ESRI multiresolution aerial imagery. Slope values are extracted to these point locations from the SRTM slope raster. Using the extracted slope values, we have identified the upper limit of slope (=20%) in the geospatial model. The details are presented in the results section.

In the first iteration, the geospatial model chose grids with the highest drainage category (D = 7) (which corresponds to very poorly drained soil) and zero slope (S = 0) and accumulated area covered by those grids was estimated. The accumulated area was then compared with the target tile drainage area from the USDA census. If the accumulated area of selected grids was less than the census, the geospatial model chose grids based on the highest drainage category (D = 7) and slope less than or equal to the next lowest slope value (S + 1). That is, an increment to the slope was applied by 1%. If the geospatial model reached the upper limit of slope (i.e., S = 20) and the accumulated area of selected grids was less than the census, then grids with drainage category greater than or equal to the next highest drainage category (D = 6) were selected. In that case, the model set the slope back to zero (S = 0). An increment to the slope was made if the accumulated area was less than the target tile drainage area from the census. The model repeated these steps until the estimated tile drainage area exceeded the USDA census tile drainage area. When the estimated tile drainage area exceeded the USDA census, the model reinitialized with the current drainage category and previous slope. With a slope increment of 0.1, the model estimated absolute error between the estimated tile drainage area and the USDA census to the current slope. A final county-level tile drainage map was generated by the model with current drainage category, and the slope corresponds to the least error (Supplementary Fig. S1). When the model finished creating tile drainage maps for all counties, county maps were mosaicked together to generate a seamless 30-m U.S tile drainage map or AgTile-US using ESRI ArcMap.

The generated AgTile-US map has a spatial resolution of 30-m, which is equivalent to the NLCD land cover. Based on the USDA farms and land in farms summary (2018), the average farm size is 443 acres. Thus, the AgTile-US tile drainage map provides adequate accuracy to identify field-level tile drainage over the U.S. However, the model presented here highly depends upon tile drainage area estimates from the USDA census and SSURGO soil data. Any uncertainty in these datasets will lead to inaccuracies in the model estimated tile drainage map.

## Data Records

The generated tile drainage spatial data (AgTile-US^37^) extends from 65° 20′ 46.49″ W to 127° 53′ 13.65″ W and 22° 51′ 41.18″ N to 51° 36′ 16.74″ N, which covers the entire CONUS. The tile drainage data and its quality details are freely available to the public in GeoTIFF format and NetCDF format through an unrestricted, public repository (Figshare^37^). The data are available at 30-m resolution in binary format, where 0 represents undrained agricultural land and 1 represents tile- drained agricultural land. In the AgTile-US data, each 30-m grid identified as tile drained is entirely tile drained. The data deliver a static representation of U.S. tile drainage for the year 2017. The data quality information is an estimate of the accuracy of the AgTile-US based on 16000 ground-truth point and is presented in next section. Upon the availability of new data, the repository will be updated with a newer version of the tile drainage map.

## Technical Validation

**Fig. 3:**
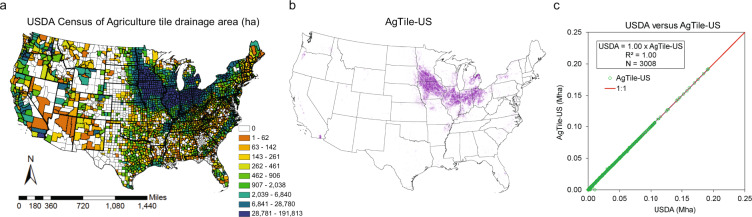
Comparison of USDA Census of Agriculture tile drainage area and AgTile-US estimates. (**a**) USDA county-level tile drainage area (ha), (**b**) the AgTile-US tile drainage data at 30 m, In (**b**), magenta color indicates grids with tile drainage, (**c**) scatterplot of USDA census and AgTile-US tile drainage area (Mha).

The spatial distribution of the tile drained area in the AgTile-US data is consistent with USDA 2017 county-level census because the geospatial model was constrained by county-level census estimates (Fig. 3). Based on the AgTile-US spatial map, most of the major tile-drained croplands across the CONUS are located in the north-central U.S., especially in Iowa, Illinois, Minnesota, Indiana, Ohio, and Michigan. Regions, including southern California, eastern Wisconsin, north-western New York, and parts of Arkansas, Missouri, and Oregon, are also identified as tile drained croplands. Even though the USDA county-level estimate was used to constrain the AgTile-US tile drainage mapping by the geospatial model, the actual tile drained area identified in the AgTile-US data is −0.35% lower for the CONUS (Table 1). The AgTile-US shows higher underestimation of tile drainage over the croplands of New Mexico (−79.06%), West Virginia (−42.38%), Mississippi (−15.90%), Oklahoma (−14.06%), and Texas (−11.53%) (Table 1). However, the total tile drained area of these states is less than 0.11 Mha. The underestimation of tile drainage by the AgTile-US is mainly due to underrepresentation of croplands in NLCD land cover 2016. Also, the USDA census reported tile drainage area for non-croplands. For instance, the tile drainage estimate from the USDA 2017 census for the Guadalupe county of New Mexico is 10602.8 ha, whereas the total cropland area based on the census is only 5386.8 ha. For the same county, the cropland area based on NLCD 2016 is 796.5 ha. A comparison of cropland from USDA county-level census 2017 and NLCD 2016 indicate an underrepresentation of croplands by −92.00%, −27.45%, and −18.15%, for West Virginia, New Mexico, and the CONUS, respectively. Since the geospatial model identified tile drained area within NLCD cropland, the underestimation of tile drainage area by the AgTile-US can be attributed to the underrepresentation of croplands in the NLCD 2016. However, for the heavily tile drained north-central U.S., the percentage difference in tile drained area between the model estimate and USDA census is less than −0.25% (Table 1).

**Table 1:** State wise tile-drained area estimates from USDA and AgTile-US for the year 2017.

State	Tile drained area (ha)		Difference (%)	State	Tile drained area (ha)		Difference (%)
	USDA	AgTile-US			USDA	AgTile-US	
Iowa	5,708,945	5,706,831	−0.04	Oklahoma	43,031	36,983	−14.06
Illinois	3,833,811	3,836,371	0.07	Virginia	42,571	40,408	−5.08
Minnesota	3,266,234	3,264,744	−0.05	Georgia	40,196	38,747	−3.6
Indiana	2,572,515	2,572,162	−0.01	Wyoming	35,090	35,657	1.62
Ohio	2,183,253	2,178,405	−0.22	Colorado	31,355	30,418	−2.99
Michigan	1,225,734	1,225,235	−0.04	Utah	31,175	29,803	−4.4
Missouri	453,346	450,114	−0.71	Idaho	29,592	29,930	1.14
Wisconsin	397,952	396,948	−0.25	Louisiana	26,518	26,245	−1.03
New York	348,537	346,233	−0.66	Alabama	24,840	24,759	−0.33
South Dakota	264,949	265,357	0.15	Maryland	18,370	16,973	−7.6
California	253,260	254,011	0.3	West Virginia	16,487	9,500	−42.38
Nebraska	223,968	224,583	0.27	New Mexico	13,943	2,919	−79.06
Kentucky	184,419	176,125	−4.5	Vermont	13,096	13,104	0.06
Arkansas	171,879	168,358	−2.05	New Jersey	10,998	10,968	−0.28
Oregon	127,125	124,760	−1.86	Mississippi	10,025	8,431	−15.9
Pennsylvania	125,337	123,859	−1.18	Maine	5,435	5,359	−1.41
Kansas	118,707	118,433	−0.23	Montana	4,919	4,887	−0.66
North Dakota	112,531	116,146	3.21	Delaware	4,619	4,610	−0.19
Texas	110,788	98,018	−11.53	Nevada	3,502	3,495	−0.2
North Carolina	110,363	107,704	−2.41	Massachusetts	1,461	1,442	−1.31
Washington	76,758	73,498	−4.25	New Hampshire	1,119	1,123	0.34
Florida	51,186	51,120	−0.13	Arizona	1,104	1,224	10.85
Tennessee	43,558	40,574	−6.85	Connecticut	929	946	1.86
South Carolina	43,434	42,926	−1.17				
Total U.S.					22,418,966	22,340,442	−0.35

**Fig. 4:**
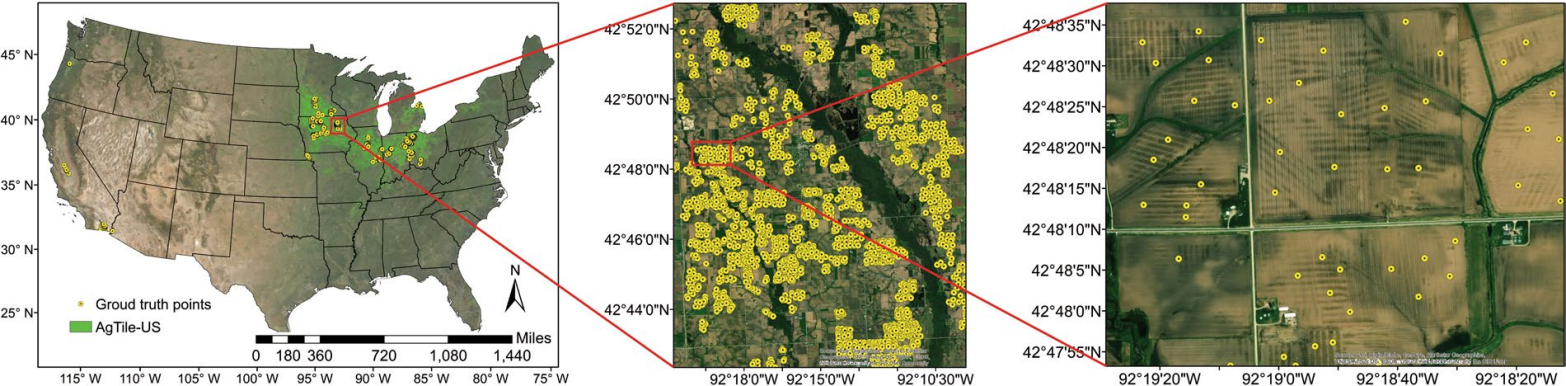
Spatial distribution of tile drainage ground-truth data (16000 points) used for the accuracy assessment. These point data were manually identified from ESRI multi-resolution (15 m, 1 m, 0.6 m, and 0.3 m) aerial imagery basemap (sources include Esri, DigitalGlobe, Earthstar Geographics, CNES/Airbus DS, GeoEye, USDA FSA, USGS, Aerogrid, IGN, IGP, and the GIS User Community; Acquirement dates of imagery ranges from 10/2/2012 to 4/23/2017 which is identified using ESRI ArcMap identification tool from the imagery basemap).

Generally, the usefulness of scientific data mainly depends upon the level of accuracy and the quality of validation data. Most of the previous studies identified tile drained croplands at a smaller scale (field, catchment, or watershed scale) than the present study and employed GIS-based, remote-sensing-based or data-intensive methods like machine learning approaches^25,29,32^ to achieve an overall accuracy of 79% to 86%. The availability and the spatial extent of validation data is the major constraint to these studies. Thus, the present study manually identified 16000 tile drainage ground-truth points across ten states of the CONUS from the ESRI multi-resolution (15 m, 1 m, 0.6 m, and 0.3 m) aerial imagery basemap (Fig. 4). The number of tile drainage ground-truth points identified depends upon the presence of a clear tile drainage signature at field-level. As indicated by *Tetzlaff et al*.^32^, the presence of vegetation and the acquisition time of aerial imagery after a rainfall event make this task difficult. So, we limited the tile drainage ground-truth identification mostly to fallow croplands where the tile drainage signature is easily detectable. The points are randomly placed in a field when a tile drainage signature is detected (Fig. 4). Simple random sampling method is used as it is the best method of sampling and each member of the population has an equal probability of being selected^38^. Slope and soil drainage categories were extracted for these 16000 points, and based on that we constrained the upper limit of slope in the model before mapping tile drainage.

**Fig. 5:**
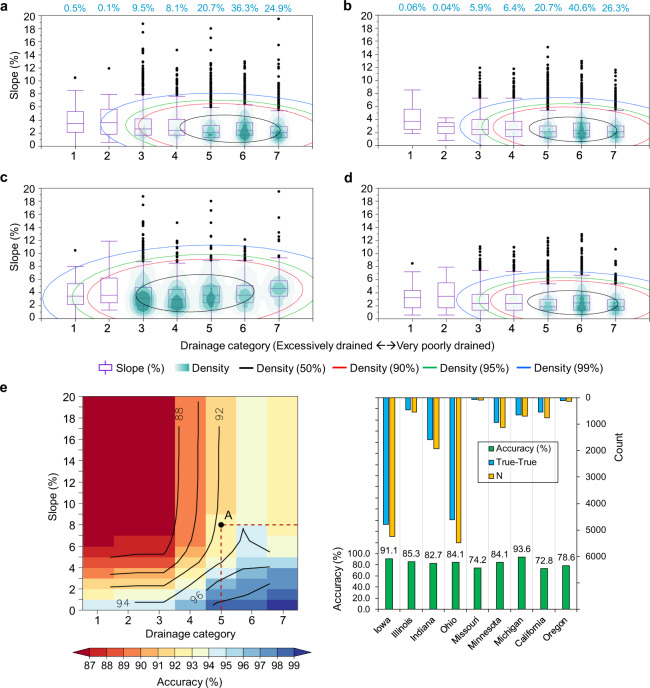
Verification of AgTile-US tile drainage data with 16000 ground truth points. **(a)** Distribution of 16000 tile drainage ground-truth points on drainage category-topographic slope two-dimensional distribution space, **(b)** As in **(a)**, but for AgTile-US tile drainage data, **(c)** As in **(b)**, but for ‘false’ AgTile-US grids when compared to ground truths (False-True), **(d)** As in **(b)**, but for valid or ‘true’ AgTile-US (True-True), In **(a–d)**, turquoise color grading over the box and whisker plot and number in turquoise color over the panel (**a,b**) indicate the data density in each drainage category and colored ellipse indicate the data distribution density (%), **(e)** AgTile-US accuracy distribution on drainage category-topographic slope two-dimensional distribution space based on ground truth points, In **(e)**, black line indicates isoline of accuracy or accuracy contour, **(f)** State-wise summary of accuracy assessment for selected nine states. In **(f)**, N indicates the total number of ground truth identified. In (**a**–**e**) x-axis indicate soil drainage categories, 1-Excessively drained, 2-Somewhat excessively drained, 3-Well drained, 4-Moderately well-drained, 5-Somewhat poorly drained, 6-Poorly drained, and 7-Very poorly drained.

To validate the AgTile-US tile drainage data with ground truth points, we plotted ground-truth points on drainage category-topographic slope two-dimensional distribution space (Fig. 5a–d) and its statistics are presented in Table 2. Figure 5a–d and Table 2 provides the information on the distribution of slope for each drainage category. For instance, the distribution of 16000 tile drainage ground-truth points on drainage category-topographic slope two-dimensional distribution space is presented in Fig. 5a, and it is clear from the figure that most (36.3%) of the tile drained croplands have poorly drained soil (category 6). Moreover, the data presented in Fig. 5a indicates a very small presence (0.5%) of tile drainage in excessively drained croplands (category = 1) and croplands with up to 10% slope. Over 50 percent of the ground-truth points are located over drainage categories greater than or equal to four and within 4.0% slope, and a slope of 8.76% or less covers 99 percent of the ground-truth points. Also, for the poorly drained category, 25% of the tile drained croplands are within 1.67% slope and a slope within 3.58% covers 75% of tile drained croplands (Table 2).

**Table 2:** Validation statistics for the AgTile-US. In Table 2, GT represents statistics for ground-truth points based on data presented in Fig. 5a and AgTile-US represents statistics for the AgTile-US based on data presented in Fig. 5b.

Drainage Category	25th Quantile slope (%)		75th Quantile slope (%)		Median slope (%)		Data Density (%)	
	GT	AgTile-US	GT	AgTile-US	GT	AgTile-US	GT	AgTile-US
1	2.12	2.63	5.17	5.07	3.44	3.72	0.5	0.06
2	1.86	1.86	5.43	3.53	3.58	2.96	0.1	0.04
3	1.67	1.67	4.17	3.95	2.64	2.46	9.5	5.9
4	1.67	1.58	4.12	3.77	2.42	2.42	8.1	6.4
5	1.32	1.32	3.17	3	2.12	2.12	20.7	20.7
6	1.67	1.32	3.58	3.44	2.42	2.35	36.3	40.6
7	1.32	1.32	3	3	2.12	2.12	24.9	26.3

To compare the AgTile-US with ground-truth points, we used 21582 random samples from AgTile-US (Fig. S2). We plotted values of slope and soil drainage category identified for 21582 random samples (used simple random sampling technique in ESRI ArcMap) on drainage category-topographic slope two-dimensional distribution space (Fig. 5b). The geospatial model captured the tile drainage ground-truth distribution characteristics (compare Fig. 5a,b, and Table 2). Identical to ground-truth points, most (40.60%) of the grids in the AgTile-US that identified as tile drained are in poorly drained croplands, and about 99 percent of the tile drained grids are within 7.77% slope. For the poorly drained category, 25% of the tile drained croplands are within 1.67% slope and a slope of 3.44% covers 75% of tile drained croplands (Table 2). The density ellipses in Fig. 5b are slightly contracted along the major and minor axis. This is because in the geospatial model, we set higher priority for poorly drained soil with low slopes. We have also extracted the AgTile-US grid values to the ground-truth points and separated the points into True-True (drained in AgTile-US and in ground-truth) and False-True (undrained in AgTile-US and drained in ground-truth), and plotted each distribution on drainage category-topographic slope two-dimensional distribution space (Fig. 5c,d). The AgTile-US failed to identify tile drainage at about 13.97% of ground-truth locations, and most of these points are in marginal drainage categories (category = 3–5). However, the True-True points (Fig. 5d), which sum up to 86.03% of total points, show a strong agreement to the ground-truth distribution (Fig. 5a).

To examine the influence of soil drainage category and topographic slope on the AgTile-US accuracy, we extended our analysis using 16000 ground-truth points. The accuracy distribution of AgTile-US on drainage category-topographic slope two-dimensional space is depicted in Fig. 5e. The AgTile-US tile drainage data indicated higher accuracy (greater than 98%) over croplands with very poorly drained and poorly drained soils and slope less than or equal to one. The accuracy of AgTile-US consistently decreases as we include grids with higher slope and increasing soil drainage. For instance, point ‘A’ in Fig. 5e indicates an accuracy of 92 to 93% (area under red dashed lines). It includes grids with drainage category from seven to five and slope from 0 to 8%. Our geospatial approach to identify tile drainage has higher accuracy (greater than 90%) over croplands with any poorly drained soils (category > = 5) and slope up to 20%, or croplands with any well-drained soils (category <5) and less than 2% slope. Also, the accuracy distribution presented in the Fig. 5e can be used to extract AgTile-US grids of required accuracy. The state-level summary of AgTile-US validation with ground-truth points is presented in Fig. 5f. The AgTile-US indicated higher accuracy (72.8% to 100%) over most of the heavily tile drained states of the north-central U.S. The state with the most tile drained croplands is Iowa (5.7 Mha), where we have identified 5253 ground truth points, and the AgTile-US showed 91.1% accuracy. To explore this underperformance, a comparison of the ground-truth points and NLCD 2016 cropland showed that 91 out of 16000 (0.57%) points are not in croplands. A comparison of NLCD 2016 cropland area with county-level cropland area estimate from the Census of Agriculture 2017^1^ indicated a good agreement with R^2^ = 0.93 and RMSE 1.82 Mha. Overall, NLCD 2016 has 18% less cropland when compared to the USDA census estimate. This indicates an added uncertainty due to using NLCD 2016 to define the crop mask. To address this uncertainty we repeated the AgTile-US methodology with the USDA CropScape crop data layer^39^. However, the overall accuracy of mapped tile drainage area using the USDA CropScape was the same as with the NLCD crop layer. Additionally, we performed a sensitivity analysis to evaluate the effect of DEM uncertainty on AgTile-US accuracy. Analysis indicated nearly similar accuracy (only ~2% lower) of AgTile-US with new DEM (a 30-m resolution DEM resampled from 90-m SRTM-DEM). The SRTM derived DEM used in this study has good accuracy (with a CONUS scale mean error of 0.16 m) to produce quality topographic slope^40,41^.

Furthermore, we constrained our geospatial model with the USDA 2017 county-level tile drainage estimate. The most significant sources of uncertainty in our tile drainage area estimate can be attributed to uncertainty in USDA tile drainage census estimates and NLCD 2016 cropland. According to the Census of Agriculture, 2017 final report^1^, there is the potential for error within the census estimate due to the adjustments for nonresponse, undercoverage, misclassification, calibration, and integerization. The average error in tile drainage estimates based on the Census of Agriculture 2017 is 19.8%. However, we consider the USDA tile drainage census estimate to be the best reliable tile drainage data since it comes from a federally published national agriculture database. Unavailability of tile drainage ground truth points over vegetated croplands and uncertainty in croplands without tile drainage constitutes the primary limitations of the validation approach we adopted.

The AgTile-US agreed well with ground-truth points and indicated an overall accuracy of 86.03% for the CONUS with an omission error of 13.97%. The amount of ground-truth points used in this study were sufficient to conclude the accuracy of AgTile-US to 86.03% (Fig. S3). Despite some remaining uncertainty, a 30-m resolution tile drainage data generated using the geospatial model and input datasets outlined in this paper are of sufficient quality to be used to answer a wide variety of questions related to tile drainage and its impacts on hydrology and environment, climate, and agriculture in the U.S.

## Usage Notes

This paper presented a robust but easy-to-implement decision-tree-based geospatial model for mapping tile drainage for the U.S. using SSURGO soil drainage information and topographic slope as inputs. The tile drainage dataset was prepared by constraining the geospatial model with USDA county-level tile drainage estimates, and thresholds of slope and soil drainage properties were estimated separately for each county. The dataset was successfully analyzed and validated with 16000 ground-truth points across the CONUS. Overall, the dataset indicated 86.03% accuracy for the CONUS, which was comparable with the accuracy of tile drainage mapping at the watershed-scale reported by other studies.

In general, the spatial information of tile drainage in necessary to study the influence of subsurface drainage on plant growth, soil quality, in-stream nutrient load, and streamflow; for applications such as hydrological modeling, flood forecasting, water balance analysis, climate modeling, spatial and temporal changes in crop yield and cropping pattern, and spatial and temporal changes in evapotranspiration. Even though subsurface tile drainage in the Midwestern U.S causes severe problems of nutrient pollution and hypoxia in the surface water, especially in the Gulf of Mexico (the “dead zone”), those tile-drainage data limitation constrains researchers to scale-up impact assessment studies from watershed scale to regional scale^21^. For instance, the tile drainage spatial map is supporting an effort to improve the prediction of streamflow over the Midwestern U.S. within the NOAA National Water Model of the U.S. Other potential uses enabled by this data set include the estimation of the contribution of subsurface tile discharge to streamflow, and the modeling of agriculture nutrient transport to the Mississippi River. This dataset can also be used to estimate the effect of tile drainage on regional climate with the help of a regional climate model.

The AgTile-US tile drainage maps are presented in georeferenced GeoTIFF, and NetCDF-4 format files along with its quality levels prepared using accuracy information presented in Fig. 5e. The user has the freedom to choose the grids of required accuracy from the AgTile-US based on the quality information provided with the dataset. This dataset can be easily imported to ESRI ArcMap^34^ and any other geospatial software.

## Supplementary information

Supplementary Information

## Data Availability

The AgTile-US model R code and example R code to read dataset are provided in GitHub (https://github.com/NCAR/AgTile-US) and Figshare^37^.
