# Supplementary material for: Mapping of 30-meter resolution tile-drained croplands using a geospatial modeling approach
Source: Sci Data. 2020 Aug 5;7:257. doi: 10.1038/s41597-020-00596-x (PMC7406500; doi:10.1038/s41597-020-00596-x)
Supplement: Supplementary file 1 — Supplementary Information [file 41597_2020_596_MOESM1_ESM.docx]

**Supplemental Information:**

**Mapping of 30-meter resolution tile drained croplands using a geospatial modeling approach**

Prasanth Valayamkunnath*^a^, Michael Barlage^a^, Fei Chen^a^, David J. Gochis^a^, Kristie J. Franz^b^

^a^National Center for Atmospheric Research (NCAR), Boulder, Colorado, USA, 80301

^b^Geological and Atmospheric Sciences, Iowa State University, Ames, Iowa, USA, 50011

*Corresponding author:prasanth@ucar.edu

**Table of contents**

| Figure | Title | Page Number |
| --- | --- | --- |
| S1 | A detailed AgTile-US geospatial model flowchart. | 2 |
| S2 | Distribution of 21582 random samples for AgTile-US. | 3 |
| S3 | Distribution of ground truth sample size versus AgTile-US accuracy. Samples are collected with replacement. | 4 |


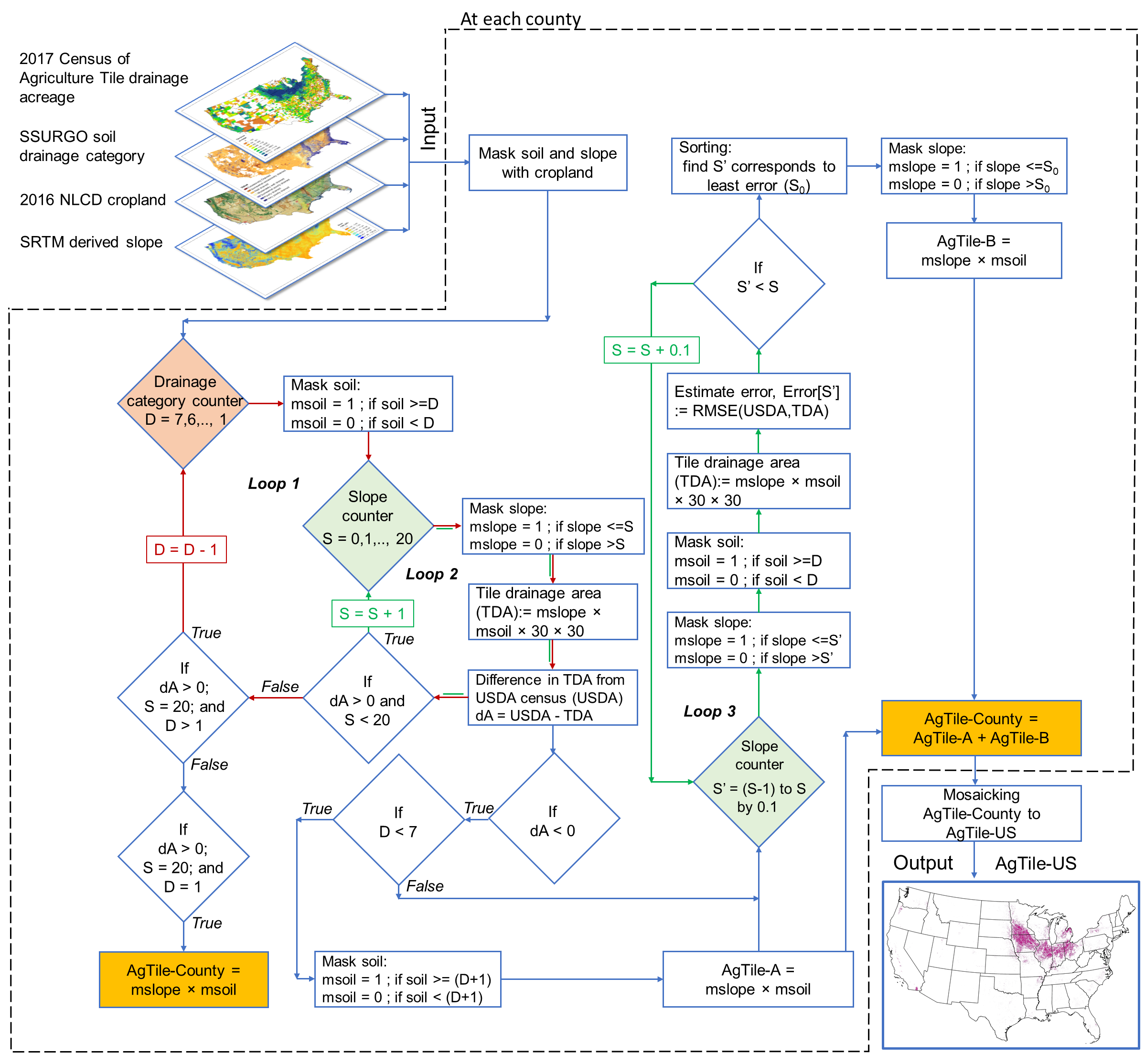


**Figure S1**. A detailed AgTile-US geospatial model flowchart.


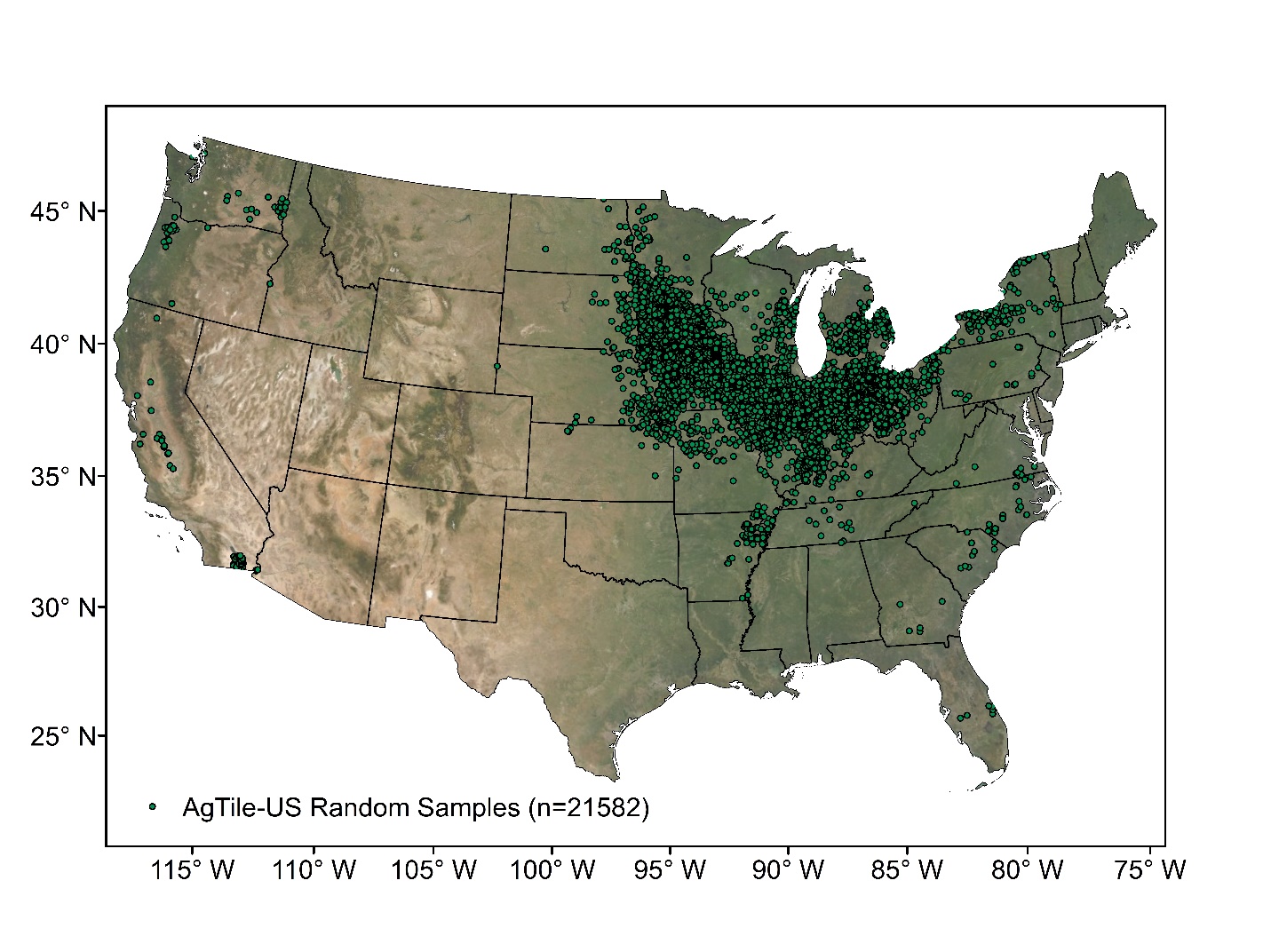


**Figure S2**. Distribution of 21582 random samples for AgTile-US.

**Figure S3.** Distribution of ground truth sample size versus AgTile-US accuracy. Samples are collected with replacement.
